# Impact of a multidisciplinary intervention on physical fitness, physical activity habits and the association between aerobic fitness and components of metabolic syndrome in adults diagnosed with metabolic syndrome

**DOI:** 10.1186/s13690-020-0399-0

**Published:** 2020-04-15

**Authors:** Angelo Tremblay, Marie-Pier Bélanger, Rupinder Dhaliwal, Paula Brauer, Dawna Royall, David M. Mutch, Caroline Rhéaume

**Affiliations:** 1 Department of Kinesiology, PEPS, Faculty of Medicine, Université Laval, Quebec, G1V 0A6 Canada; 2 Metabolic Syndrome Canada, Kingston, Ontario Canada; 3 Department of Family Relations & Applied Nutrition, University of Guelph, Guelph, ON Canada; 4 Department of Human Health and Nutritional Sciences, University of Guelph, Guelph, ON Canada; 5 Centre de recherche de l’Institut universitaire de cardiologie et de pneumologie de Québec and Department of Family Medicine and Emergency Medicine, Faculty of Medicine, Université Laval, Quebec, Canada

**Keywords:** Plasma glucose, Waist circumference, Blood pressure, VO_2_max, Exercise, Lifestyle

## Abstract

**Background:**

Metabolic syndrome (MetS) is a health disorder characterized by metabolic abnormalities that predict an increased risk to develop cardiovascular disease (CVD) and type 2 diabetes (T2DM). It can be resolved, and its complications reduced, by lifestyle interventions offered in primary care. The objectives of this study were to evaluate the impact of the exercise program of the CHANGE feasibility study on physical fitness and physical activity habits, and assess associations between changes in MetS components and cardiorespiratory fitness (CRF).

**Methods:**

In this analysis of 192 of the 293 adults with MetS in the overall study, the impact on physical fitness [aerobic capacity, muscular fitness and flexibility], and non-supervised physical activities was investigated over 12 months. In the CHANGE program, aerobic capacity, muscular fitness and flexibility were assessed at baseline, after 3 months of weekly supervised exercise, and following 9 additional months during which participants had one monthly session of supervised exercise. Additionally, CRF response was also examined in relation to changes in MetS components [fasting glucose, high-density lipoprotein (HDL) cholesterol, triglycerides, blood pressure, waist circumference (WC)].

**Results:**

Fitness variables were significantly increased at 12 months with most of the improvements reached by 3 months (estimated VO_2_ max: 6 and 12%; partial curl-ups: 55 and 80%; push-ups: 50 and 100%; flexibility: 22 and 10% in men and women, respectively, *p* <  0.001). As expected, the duration and intensity of supervised aerobic physical activity increased during the first 3 months of supervision in both men and women, and remained unchanged for the duration of the program. The duration of non-supervised physical activities did not change during the program in men whereas an increase in manual work of moderate intensity was recorded in women between 3 and 12 months. In women, mean changes in WC were significantly greater among high VO_2_ max responders than low responders, between 0 and 12 months, as well as between 3 and 12 months (− 3.42 cm and − 4.32 cm, respectively, *p* <  0.05). No associations were seen with MetS components in men. Higher intensity activities were maintained by both sexes at one year.

**Conclusion:**

Patients with MetS participating in the CHANGE lifestyle program improved physical fitness and physical activity habits by three months and maintained these gains over one year. Women who achieved a greater VO_2_ max increase had greater reductions in WC compared to low VO_2_max responders.

## Résumé

**Contexte:**

Le syndrome métabolique (SMet) est un problème de santé caractérisé par des anomalies métaboliques prédisant un risque accru de développer une maladie cardiovasculaire (MCV) et un diabète de type 2 (DT2). Il peut être résolu et ses complications atténuées par des interventions axées sur le mode de vie. Les objectifs de cette étude étaient de mesurer l’impact du programme CHANGE sur la condition physique et les habitudes d’activité physique, et d’évaluer les associations entre les modifications des composantes du SMet et la capacité cardiorespiratoire.

**Méthodes:**

Dans cette analyse de 192 des 293 adultes atteints du SMet, l’impact sur la condition physique [capacité aérobie, vigueur musculaire et flexibilité] et sur les activités physiques non supervisées a été étudié pendant une période de 12 mois. Pour ce qui est du programme d’exercice, la capacité aérobie, l’endurance musculaire et la flexibilité ont été évaluées au départ, après 3 mois d’exercice hebdomadaire supervisé et après 9 mois supplémentaires au cours desquels les participants ont eu une session mensuelle d’exercice supervisé. La réponse de la capacité cardiorespiratoire a également été examinée en relation avec les modifications des composantes du syndrome métabolique (glucose à jeun, HDL-cholestérol, triglycérides, pression artérielle, tour de taille).

**Résultats:**

Les variables de la condition physique ont été significativement augmentées à 12 mois avec la plupart des améliorations atteintes à 3 mois (VO_2_ max estimée: 6 et 12%; redressements partiels: 55 et 80%; push-ups: 50 et 100%; flexibilité: 22 et 10% chez les hommes et les femmes, respectivement, *p* <  0,001). Comme prévu, la durée et l’intensité de l’activité physique aérobie supervisée ont augmenté au cours des 3 premiers mois de supervision chez les femmes et les hommes et sont par la suite demeurées inchangées jusqu’à la fin du programme. La durée des activités physiques non encadrées n’a pas changé au cours du programme chez les hommes alors qu’une augmentation du travail manuel d’intensité modérée a été enregistrée chez les femmes entre 3 et 12 mois. Chez les femmes, les changements moyens dans la circonférence de taille étaient significativement plus importants chez les bons répondeurs au VO_2_max que chez les plus faibles répondeurs, entre 0 et 12 mois, ainsi qu’entre 3 et 12 mois (− 3,42 cm et − 4,32 cm, respectivement, *p* <  0,05). Aucune association n’a été observée avec les composantes du SMet chez les hommes. Les activités d’intensité plus élevée ont été maintenues par les femmes et les hommes à un an.

**Conclusion:**

Les patients atteints de SMet participant au programme CHANGE ont amélioré leur condition physique et leurs habitudes d’activité physique après 3 mois et ont maintenu ces gains pendant un an. Les femmes qui ont eu une plus grande augmentation du VO_2_max ont aussi obtenu une plus grande diminution de la circonférence de taille par rapport à celles ayant un faible VO_2_max.

## Introduction

Metabolic syndrome (MetS) is a risk factor for cardiovascular disease (CVD) and development of type 2 diabetes (T2DM) that encompasses a limited cluster of metabolic abnormalities linked to insulin resistance that is often associated with abdominal obesity [1]. Studies have shown that physical inactivity is associated with most, if not all, components of MetS [2, 3], as well as an increased risk of CVD [4] and diabetes [5]. It has also been demonstrated that lifestyle interventions, such as diet and exercise, have the potential to improve clinically relevant outcomes [6, 7]. Recent meta-analyses of randomized controlled trials showed that MetS can be resolved and its components can be significantly reduced by diet and exercise interventions [5, 8]. Aerobic exercise training in patients with MetS is also effective in reducing several risk factors in individuals already at high risk for metabolic disease [9]. In the RESOLVE trial of individuals with MetS testing effects of endurance vs resistance exercise, while controlling diet, a more pronounced decrease in visceral fat was seen with increased intensity of either endurance or resistance exercise [10]. Evidence is also emerging that increased physical activity can be sustained over time. More than 10 years after the start of the Diabetes Prevention Program, participants still performed more moderate to vigorous physical activity (MVPA) than reference adults from the National Health and Nutrition Examination Survey (NHANES; 2003–2006) [11].

Cardiorespiratory fitness (CRF) has been documented for its inverse association with cardiovascular (CV) morbidity and mortality [12]. A growing body of epidemiological and clinical evidence demonstrates not only that CRF is a potentially stronger predictor of mortality than traditional risk factors such as diabetes, hypertension and dyslipidemia, but the addition of CRF to established risk factors can significantly attenuate the increased risk for adverse outcomes [13]. Numerous studies have demonstrated inverse associations between CRF and the risk of developing prediabetes, MetS, and T2DM [14]. The dose-response relationship observed between CRF and MetS is curvilinear in nature [15, 16]. Thus, physical activity interventions targeting the least fit individuals may have the largest benefit in reducing cardiometabolic risk.

Despite these promising results, the proportion of patients with or without T2DM who receive more than brief verbal recommendations on healthy lifestyle changes, such as increased physical activity and improved diet, from their primary care physician remains low [17]. Recently, we examined the feasibility of an intensive lifestyle program in team-based primary care and demonstrated clinically meaningful results. The MetS diagnosis was made by the family physician who was supported by a dietitian and a clinical kinesiologist who provided tailored follow-up of lifestyle changes over one year. We observed that the intervention induced favorable changes in some MetS components and a reversal/remission of MetS in 19% of patients [18]. We report here the effects of the exercise component of the intervention on aerobic and muscular fitness and non-supervised physical activity participation. In addition, we assessed the relationship between variations in aerobic fitness and the response of MetS components to the intervention.

## Methodology

### Study design and participants

The present analysis was performed with data from a prospective, longitudinal before-after demonstration study (the CHANGE feasibility study) conducted in 3 primary care clinics in Canada (Toronto, Quebec and Edmonton) that was aimed to test the feasibility and effectiveness of implementing a program based on healthy eating and graded exercise to improve MetS [18]. The details and main results of the study have been reported elsewhere [18]. Among the 293 subjects who participated in this study, 192 patients (97 males and 95 females) aged ≥18 years had complete data for fitness and physical activity participation. Written and oral informed consent was obtained from all eligible patients before inclusion. This protocol was approved by Research Ethics Boards at each of the participating universities (Toronto, Guelph, Laval and Alberta) and according to local requirements for the primary care organizations (Edmonton Oliver Primary Care Network, Edmonton; Centre de Santé et des Services Sociaux de la Capitale, Quebec City; Polyclinic Family & Specialty Medicine, Toronto). The inclusion and exclusion criteria considered in this study are presented in the Appendix. Eligible patients identified by their family doctor (FD) as having MetS were referred to the Registered Dietitian (RD) at each clinic for a tailored diet plan, based on a care map that incorporated evidence from clinical trials and principles of health behaviour change from the Integrated Behavioral Model [19], with emphasis on Mediterranean diet principles [20]. Each participant was also referred to a clinical exercise specialist for assessment of their fitness and physical activity habits, and for an individualized fitness plan that included aerobic activity, resistance training and flexibility exercises.

The intervention had two phases: weekly visits for the first 3 months, then monthly for the next 9 months. As reported by Klein et al. [21], all participants were followed by the exercise specialist who was also responsible for fitness measurements at baseline and following 3 and 12 months of supervision and for physical activity monitoring throughout the program.

### Aerobic and muscular exercise program

There were 21 prescribed fitness contacts in total per patient and the exercise program consisted of aerobic exercises using equipment such as treadmill, ergocycle, elliptic machine, stair machine and rowing machine. At the beginning of the program, it was expected that work bouts of 20–30 min could be performed at 50% maximal heart rate 3 times a week with the goal to increase the exercise duration, intensity and frequency in the first 3 months of the program. The theoretical targeted duration, intensity and frequency after 3 months was 45–50 min per session, 65–75% of maximal heart rate, and 3 to 5 sessions per week which conforms with the American College of Sports Medicine (ACSM) and the Canadian Society for Exercise Physiology (CSEP) guidelines [22, 23]. However, considering the fitness and/or time limitations of many participants, actual reported frequency of exercise was typically lower than recommended guidelines. The exercise specialists optionally recorded the type of exercise (cycling, jogging, swimming, etc.), its duration (min per session) and intensity (continuous heart rate in bpm when available), and its weekly frequency for every participant during each session of supervised exercise.

Muscular and flexibility exercises were also prescribed on a personalized basis by the exercise specialist that considered the limitations of each participant. These exercises included movements soliciting muscles of the upper and lower limbs as well as dorsal and abdominal muscles. Muscular and flexibility exercises in terms of exercise identification, number of repetitions and perceived effort (modified Borg scale [24]) were also optionally recorded.

## Measurements

### Fitness assessment

All patients underwent a fitness assessment using standardized procedures recommended by the CSEP [23]. The assessment allows the calculation of a Health Benefits value based on age and sex according to the results obtained for each test [23].

The single stage walking test described by Ebbeling et al. was used to estimate maximal oxygen consumption [25]. This submaximal aerobic fitness test requires a treadmill and a heart rate monitor for its administration. Briefly, participants completed a 4 min warm-up at 0% grade while walking at a heart rate between 50 and 70% of estimated maximal heart rate that was determined with the Karvonen equation (max heart rate (bpm) = 220 - age) [26]. After the warm-up, the participant kept the same speed for an additional 4 min at a grade of 5%, and then a record of the speed, steady-state heart rate, blood pressure and perception of effort (Modified Borg Scale) was collected during the final 30 s of the last two minutes. The equation validated by Ebbeling et al. was used for the estimation of VO_2_max (ml/kg/min). To obtain the aerobic fitness score, the estimated VO_2_max was multiplied by 10.

#### Muscular fitness tests

The kinesiologist also assessed muscular endurance with the measurement of partial curl-ups and push-ups, as described by the CSEP [23]. For the first test, participants were in supine position with their knees at 90 degrees. Arms were at their side, palms facing down with the middle finger touching the piece of masking tape. Then, participants did slow, controlled curl-ups to touch the second piece of a masking tape positioned 10 cm apart by lifting the shoulder blades off the mat at a standardized rate. The maximal number of partial curl-ups (up to 25) in one minute without pausing was counted. For the second test, starting in a prone position with their hands under the shoulders, participants raised their body by straightening the elbows and using their knees as the pivot point. Then, they returned to the starting position while maintaining adequate technique. The maximal number of push-ups performed consecutively without rest in one minute was counted as the score.

#### Flexibility test

Flexibility was evaluated using a sit and reach protocol [23]. The participants sat with legs fully extended, without shoes, and the soles of their feet placed flat against the flexometer. Keeping their knees fully extended, arms evenly stretched, and palms down, participants were instructed to bend and reach forward in a controlled manner pushing the sliding marker along with the scale with their fingertips as far forward as possible. The maximal flexion position had to be held for approximately two seconds. The test was repeated twice and the maximal distance (cm) was recorded.

### Non-supervised exercise

In addition to the records completed by the exercise specialist, a subgroup of participants from the Quebec site completed an exercise diary, weekly, then monthly at 3 to 12 months, in which the type of exercise and the mean duration of unsupervised exercise performed outside the training centres were noted. In accordance with the categories described by Bouchard et al. [27], the participant diary included four types of non-supervised exercise: leisure activities and sports in a recreational environment (baseball, golf, cycling < 10 km/h, etc.); manual work at moderate intensity (snow shovelling, loading and unloading goods, etc.); leisure and sports activities of higher intensity (not competitive) (cycling > 15 km/h, dancing, walking > 6 km/h, etc.) and intense manual work or high intensity sport activities (jogging and running > 9 km/h, racquetball, badminton, hiking, etc.).

### Assessment of components of the MetS

The program included visits with the FD at baseline and at 3, 6, 9 and 12 months for a review of participants’ medical profile related to MetS. The diagnosis of MetS was based on reference values proposed by the international harmonized definition for blood pressure, waist circumference (WC), plasma glucose and plasma lipids (triglycerides and high-density lipoprotein (HDL) cholesterol) [28]. These blood variables were part of the initial bloodwork in the routine patient care. As described elsewhere [18], they were used for both clinical and research purposes. The measurement of MetS components was performed according to standard procedures in the three primary care clinics participating in this study.

### Statistical analyses

Baseline characteristics were compared between groups (men and women) using a t-test for all variables. For all continuous outcomes, data at each time point are presented as raw mean and standard error (SE) and were separately assessed by sex. For physical fitness and MetS variables, the mean values were compared at baseline, 3 and 12 months using a linear mixed model analysis after having verified the normality of score distribution. It is to be noted that the analysis for values of plasma triglycerides was done with log transformed data. The sample of subjects was also divided into tertiles for baseline estimated VO_2_max, as well as changes in VO_2_ max, to evaluate the potential impact of VO_2_max on the response of MetS components to the lifestyle intervention. The difference between measurements of MetS components at baseline, 3 and 12 months were used to evaluate the relationship between CRF and cardiometabolic health. For the non-supervised exercise, the mean daily time allocated to each exercise category was compared at baseline, 3 and 12 months using a two-way ANOVA. A Tukey’s post-hoc analysis was performed when a significant F value was obtained. A *P*-value of < 0.05 was considered to indicate statistical significance. All analyses were performed using SAS Version 9 (SAS Institute, Inc., Cary, NC, USA).

## Results

### Patient characteristics

As previously described [18], we aimed to enrol a total of 300 participants from the three sites involved in the study. This sample size was expected to provide a 95% chance of estimating the true reversal rate within 5%, assuming a reversal rate of 25% or less and a random distribution of the contacts with the dietitian and the exercise specialist. Of the 293 enrolled patients, 253 had complete MetS lab data at 12 months and of these 192 (76%) had complete data on fitness at 12 months. Baseline patient characteristics are presented in Table 1. As expected, height, weight and estimated VO_2_max were significantly different between men and women (*p* <  0.001). The median number of fitness visits was 16 (range = 10 to 20), indicating that the median patient attended 76% of the 21 prescribed fitness visits over the 12 months.

**Table 1:** Baseline patient characteristics

	All patients (*n* = 192)	Men (*n* = 97)	Women (*n* = 95)	*P* values
Age (years)	59.84 ± 0.66	58.96 ± 0.94	60.75 ± 0.91	0.1730
Current smoker (n (%))	15 (7.81)	6 (6.19)	9 (9.47)	0.3996
Height (meters)	1. 69 ± 0.01	1.75 ± 0.01	1.62 ± 0.01	< 0.0001
Weight (kg)	88.60 ± 1.03	94.84 ± 1.24	82.22 ± 1.38	< 0.0001
Waist circumference (cm)	106.29 ± 0.66	107.99 ± 0.84	104.54 ± 0.99	0.0085
BMI (kg/m^2^)	31.04 ± 0.25	30.79 ± 0.34	31.29 ± 0.38	0.3223
VO_2_max (ml^.^kg^-1.^min^− 1^)	32.81 ± 0.50	38.06 ± 0.52	27.46 ± 0.39	< 0.0001

Data are mean ± standard error (SE) or n (%). *BMI* Body Mass Index

*Note: P* values based on *t* test/ANOVA for all variables

### Aerobic fitness, muscular and flexibility test

The program was theoretically expected to permit the achievement of reference guidelines (22, 23) for duration, intensity and frequency of exercise within the first 3 months. Prescriptions were adjusted by the kinesiologists according to the fitness level of participants, as well as their personal circumstances and lifestyle. In this regard, Table 2 shows variations over time of personalized targets and measured values during the program. Since most of the time available to exercise specialists at baseline (week 0), month 3 (week 12) and month 12 was allocated to fitness testing and measurements, we present data collected at weeks 1 and 11, as well as at month 11, to minimize missing values in aerobic exercise duration and intensity during supervised sessions for each phase of the intervention. As expected, targeted and measured exercise duration and intensity aligned with reference values at the beginning of the program. Table 2 also shows that exercise supervision promoted a significant increase in aerobic exercise duration and intensity at the beginning of the intervention that was maintained at the end of the program.

**Table 2:** Personalized targets and measured values of aerobic exercise duration and intensity for study participants

	Time	Aerobic exercise							
		Duration (min)				Intensity (bpm)			
		*N*	Targeted	*N*	Measured	*N*	Targeted	*N*	Measured
Men	Week 1	84	25.73 ± 0.52^a^	84	26.12 ± 0.54^a^	83	111.28 ± 1.99^a^	83	117.58 ± 1.78^a^
	Week 11	72	34.28 ± 0.87^b^	72	34.46 ± 0.90^b^	71	119.37 ± 2.45^b^	72	126.76 ± 2.47^b^
	Month 11	65	33.85 ± 0.97^b^	65	32.69 ± 1.07^b^	65	117.38 ± 2.42^b^	65	125.74 ± 2.45^b^
	*P* values†		< 0.0001		< 0.0001		< 0.0001		< 0.0001
Women	Week 1	84	25.65 ± 0.62^a^	83	26.2 ± 0.64^a^	84	110.11 ± 1.85^a^	82	115.34 ± 1.82^a^
	Week 11	66	33.23 ± 0.95^b^	66	33.41 ± 0.98^b^	66	116.00 ± 2.25^b^	66	120.47 ± 2.19^b^
	Month 11	78	32.73 ± 0.85^b^	78	31.99 ± 0.96^b^	78	114.53 ± 1.92^b^	78	122.60 ± 2.05^b^
	*P* values†		< 0.0001		< 0.0001		< 0.0001		< 0.0004

Values are mean ± standard error (SE)

† *P* values based on linear mixed model analysis within each group (men and women). Values within each column with different superscript letter (a, b) are significantly different (*p* <  0.05)

As explained in the results section, exercise duration and intensity were measured at weeks 1 and 11 and month 11 to represent changes over time (instead of months 0, 3 and 12) to minimize missing values

Aerobic and muscular fitness and flexibility characteristics in men and women are shown in Table 3. As expected, the exercise intervention led to an increase in speed, VO_2_max, aerobic fitness score, and a decrease in steady-state heart rate and health benefit zone scores with no significant difference between month 3 and month 12. However, women increased their walking speed at each time point (*p* <  0.0001). The steady-state heart rate did not significantly change during the intervention for men, while it decreased at 3 months in women to ultimately return to baseline values by the end of the study. Table 3 also shows that improvements in both sexes were observed in muscular fitness as well as flexibility parameters. Specifically, partial curl-ups, push-ups and flexibility significantly increased at month 3 with no significant improvement between 3 and 12 months. However, flexibility significantly increased throughout the intervention in men and reached its maximum value at the end of the intervention.

**Table 3:** Aerobic fitness, muscular and flexibility characteristics for study subjects

	Men (*N* = 97)				Women (*N* = 95)			
	Baseline	Month 3	Month 12	*P* Values†	Baseline	Month 3	Month 12	*P* Values†
Speed (mph)	3.2 ± 0.1^a^	3.6 ± 0.1^b^	3.7 ± 0.1 ^b^	< 0.0001	2.5 ± 0.1^a^	3.0 ± 0.1^b^	3.2 ± 0.1^c^	< 0.0001
Steady-state heart rate (bpm)	120 ± 2	119 ± 2	122 ± 2	0.1635	123 ± 2 ^a^	119 ± 2 ^b^	122 ± 2^a^	0.0004
VO_2_max (ml^.^kg^-1.^min^−1^)	38.1 ± 0.6 ^a^	40.5 ± 0.6 ^b^	40.4 ± 0.6 ^b^	< 0.0001	27.5 ± 0.4 ^a^	30.1 ± 0.4 ^b^	30.8 ± 0.4 ^b^	< 0.0001
Aerobic fitness score (unit)	381.5 ± 5.5^a^	404.8 ± 0.6 ^b^	403.8 ± 5.5 ^b^	< 0.0001	274.6 ± 4.6 ^a^	301.3 ± 4.6 ^b^	308.4 ± 4.6 ^b^	< 0.0001
Health benefit zone - (aerobics) (0 to 4)^§^	2.0 ± 0.1^a^	1.6 ± 0.1 ^b^	1.6 ± 0.1 ^b^	< 0.0001	3.8 ± 0.1 ^a^	3.3 ± 0.1 ^b^	3.1 ± 0.1 ^b^	< 0.0001
Partial curl-ups (repetition 0 to 25)	11 ± 1 ^a^	16 ± 1 ^b^	17 ± 1 ^b^	< 0.0001	5 ± 1 ^a^	8 ± 1 ^b^	9 ± 1 ^b^	< 0.0001
Health benefit zone - (Partial curl-ups) (0 to 4)^§^	3.5 ± 0.2 ^a^	2.6 ± 0.2 ^b^	2.4 ± 0.2 ^b^	< 0.0001	4.1 ± 0.2 ^a^	3.7 ± 0.2 ^b^	3.4 ± 0.2 ^b^	< 0.0001
Push-ups (repetition)	8 ± 1 ^a^	11 ± 1 ^b^	12 ± 1 ^b^	< 0.0001	4 ± 1 ^a^	7 ± 1 ^b^	8 ± 1 ^b^	< 0.0001
Health benefit zone - (Push-ups) (0 to 4)^§^	3.8 ± 0.12 ^a^	3.3 ± 0.2 ^b^	3.1 ± 0.2 ^b^	< 0.0001	4.0 ± 0.1 ^a^	3.6 ± 0.1 ^b^	3.4 ± 0.1 ^b^	< 0.0001
Flexibility (cm)	16.5 ± 1.0 ^a^	19.0 ± 1.0 ^b^	20.2 ± 1.0^c^	< 0.0001	23.0 ± 1.0 ^a^	24.9 ± 1.0 ^b^	25.3 ± 1.0 ^b^	0.0003
Health benefit zone - (Flexibility) (0 to 4)^§^	4.0 ± 0.1 ^a^	3.7 ± 0.1 ^b^	3.5 ± 0.1 ^b^	< 0.0001	3.9 ± 0.2 ^a^	3.5 ± 0.2 ^b^	3.5 ± 0.2 ^b^	< 0.0001

Data are mean ± standard error (SE). ^§^ A lower score indicates a better performance. † *P* values obtained by comparing differences in the variable values within each group (men, woment) by using a linear mixed model analysis

Table 4 presents VO_2_max characteristics of subjects classified according to tertiles of baseline VO_2_max. As expected, VO_2_max significantly increased over time in each tertile for both men and women. Additionally, there were no significant time × tertile interactions, indicating that subjects responded similarly to the program in each tertile. Furthermore, most of the increase in VO_2_max in both sexes was achieved within the first three months of the intervention.

**Table 4:** Variations of VO_2_ max overtime in subjects classified according to tertiles of baseline VO_2_

		VO_2_ max (ml^.^kg^-1.^min^−1^)			
		All subjects	Low (*n* = 33)	Medium (*n* = 32)	High (*n* = 32)
Men (*n* = 97)	Baseline	38.1 ± 0.5^a^	32.7 ± 0.3^a^	37.8 ± 0.2^a^	43.9 ± 0.6^a^
	3 months	40.5 ± 0.6^b^	34.6 ± 0.3^b^	40.2 ± 0.3^b^	46.7 ± 0.7^b^
	12 months	40.4 ± 0.6^b^	34.5 ± 0.4^b^	40.2 ± 0.3^b^	46.4 ± 0.6^b^
			(*n* = 31)	(*n* = 32)	(*n* = 32)
Women (*n* = 95)	Baseline	27.5 ± 0.4^a^	23.4 ± 0.3^a^	27.1 ± 0.2^a^	31.7 ± 0.4^a^
	3 months	30.1 ± 0.4^b^	25.7 ± 0.4^b^	30.2 ± 0.2^b^	34.4 ± 0.3^b^
	12 months	30.9 ± 0.4^b^	26.5 ± 0.3^b^	30.7 ± 0.2^b^	35.1 ± 0.3^b^

Data are mean ± standard error (SE)

A two-way ANOVA with repeated measures revealed a significant effect of VO_2_ max tertiles (*P* <  0.0001) and of time (*P* <  0.0001) for each group (men and women). There were no significant time* tertiles interaction effect for men and women. Values within the same column and same gender with different superscript letters (a,b) are significantly diffirent (*P* < 0.0001)

### Metabolic syndrome

As shown in Table 5, the intervention induced a significant decrease in diastolic and systolic blood pressure and waist circumference in both men and women. Plasma triglyceride levels were also reduced in men. The response over time was similar to that of the fitness indicators, i.e. most of the benefits were observed after the first three months of intervention.

**Table 5:** Components of MetS for study subjects

Variables	Baseline	Month 3	Month 12	*p* values*
Men (*N* = 97)				
Diastolic blood pressure (mm Hg)	82.3 ± 0.8	78.1 ± 0.8	78.1 ± 0.8	< 0.0001
Systolic blood pressure (mm Hg)	135.1 ± 1.3	126.7 ± 1.3	129.6 ± 1.3	< 0.0001
Fasting blood glucose (mmol/L)	6.43 ± 0.12	6.19 ± 0.12	6.39 ± 0.12	0.0352
HDL cholesterol (mmol/L)	1.08 ± 0.02	1.09 ± 0.02	1.13 ± 0.02	0.0011
Triglyceride (mmol/L)	2.47 ± 0.18	1.79 ± 0.19	1.86 ± 0.19	< 0.0001
Waist circumference (cm)	108.0 ± 0.9	105.3 ± 0.9	104.1 ± 0.9	< 0.0001
Women (*N* = 95)				
Diastolic blood pressure (mm Hg)	79.0 ± 0.9	75.6 ± 0.9	76.3 ± 0.9	0.0004
Systolic blood pressure (mm Hg)	134.0 ± 1.5	126.3 ± 1.5	129.1 ± 1.5	< 0.0001
Fasting blood glucose (mmol/L)	6.39 ± 0.14	6.25 ± 0.14	6.33 ± 0.14	0.4129
HDL cholesterol (mmol/L)	1.31 ± 0.03	1.29 ± 0.03	1.38 ± 0.03	< 0.0001
Triglyceride (mmol/L)	2.01 ± 0.09	1.83 ± 0.09	1.84 ± 0.09	0.0184
Waist circumference (cm)	104.5 ± 1.0	101.8 ± 1.0	99.9 ± 1.0	< 0.0001

Data are mean ± standard error

* *p* values obtained by comparing differences in the variable value within each group (men, women) by using a linear mixed model analysis

There were no significant differences in mean changes for all MetS components between VO_2_max tertiles in men (data not shown). There were no significant differences in mean changes between each time point in plasma concentrations of glucose, triglycerides and HDL cholesterol as well as blood pressure between each VO_2_max tertile for women.

**Fig. 1:**
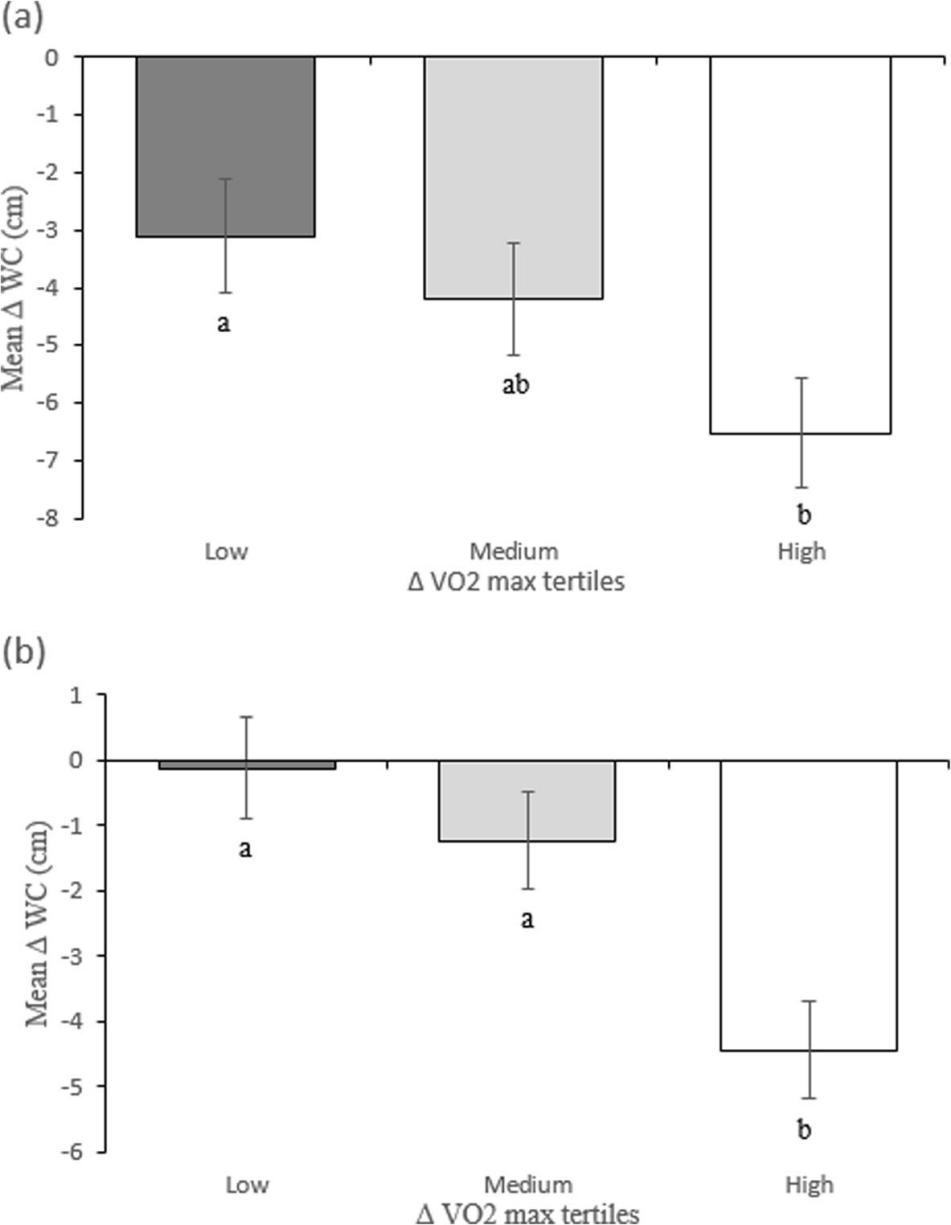
Mean changes in waist circumference (WC) between baseline and month 12 (Panel **a**) and mean changes in waist circumference (WC) between month 3 and month 12 (Panel **b**) for women according to changes in VO_2_ max tertiles. Panel A: ∆VO_2_max values were 0.24 (0.24), 2.80 (0.11), and 7.24 (0.56) (Mean ± SE) ml^.^kg^-1.^min^− 1^ for the low, medium and high tertiles, respectively. Panel B: Corresponding ∆VO_2_max values were − 1.48 (0.33), 0.54 (0.06), and 3.13 (0.35). Values within the same panel with different superscript letters (a,b) are significantly different (*P* <  0.05, Tukey-Kramer post hoc)

Figure 1 shows that mean changes in WC between baseline and month 12 (Panel A), and mean changes in WC between month 3 and month 12 (Panel B), differed significantly between tertiles of change in VO_2_max in women (*P* <  0.05 and *P* <  0.001, respectively). Mean changes in WC were significantly greater in high VO_2_max responders than low responders at both times (− 4.32 cm and − 3.42 cm, respectively, *P* <  0.05). The correlations between the change in WC and the change in VO_2_max coefficient was − 0.27 (*P* = 0.007) and − 0.28 (*p* = 0.007) between baseline and month 12 and between month 3 and month 12, respectively.

### Physical activity habits

**Fig. 2:**
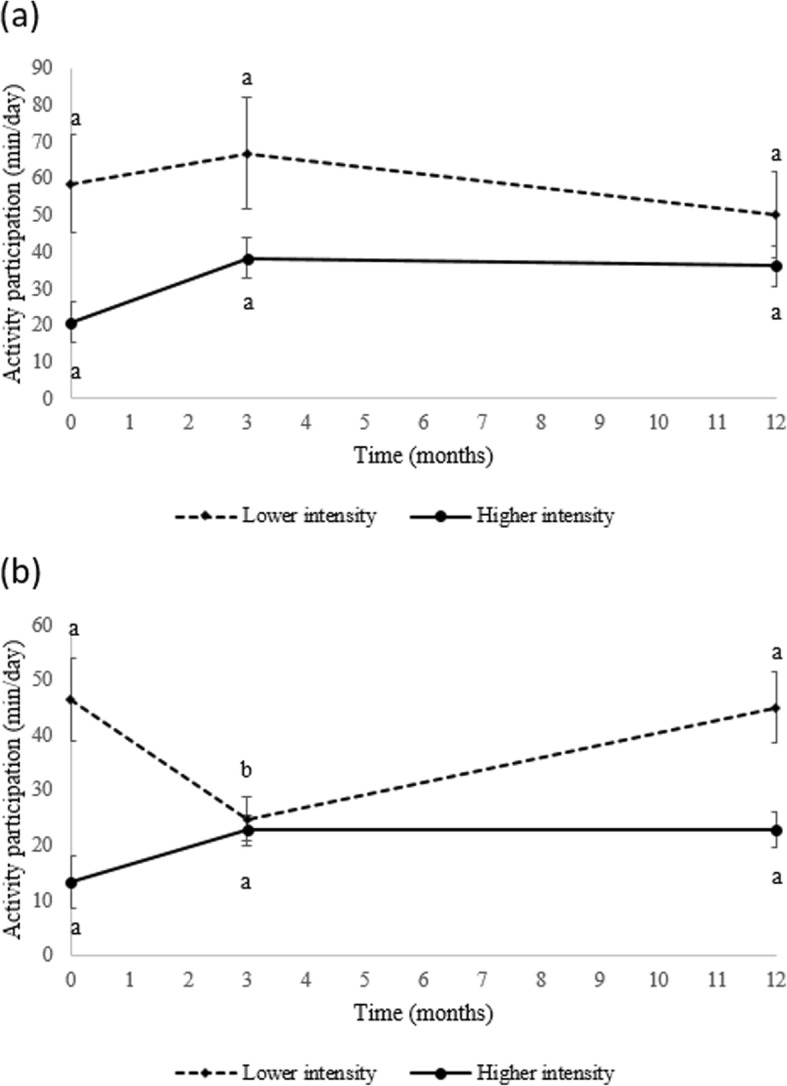
Activity participation classified in two categories of intensity for men (Panel **a**) and women (Panel **b**) in Laval University participants. Lower intensity: sum of leisure activities and sports and manual work at moderate intensity; Higher intensity: sum of leisure and sports activities of higher intensity and intense manual work, high intensity sport activities or sport competition. Values within the same line and same gender with different superscript letters (a,b) are significantly different (*P* <  0.01, Tukey-Kramer post hoc)

The average daily time spent in the four categories of activities for a subset of men (*N* = 17, baseline; *N* = 34, 3 months; *N* = 31, 12 months) when they were not supervised by the kinesiologist did not significantly change over time. In women, some variations were significant, including an increase in manual work of moderate intensity between 3 and 12 months (*N* = 38, 3 months; *N* = 34, 12 months; *P* = 0.02). To further investigate this trend, we reanalyzed the data by grouping the two categories of activities of lower intensity and comparing it to the two categories of activities of higher intensity. Interestingly, Fig. 2 illustrates that women (*N* = 20, baseline; *N* = 38, 3 months; *N* = 34, 12 months) significantly maintained the same time doing higher intensity activities during the intervention. The Pearson correlation coefficients (R) were low and not statistically significant for the association between changes in daily participation to two categories of non-supervised exercises of different intensity and changes in VO_2_max for men and women.

## Discussion

The main hypothesis that an intervention involving physicians and other health professionals would induce a significant mean increase in aerobic capacity and muscular fitness was supported by this study. Our results showed that both men and women generally increased their walking speed, VO_2_max, aerobic fitness score, partial curl-ups, push-ups and flexibility, and decreased their steady-state heart rate and health benefit zone scores with no significant difference between month 3 and month 12 despite a reduction in the frequency of kinesiologist visits during this period. The same general trend can be seen in the change of estimated VO_2_max within each VO_2_max tertile. The results of this study show more beneficial effects on WC in women who achieved a greater VO_2_max increase compared to women who had lower VO_2_max responses. Interestingly, this benefit was not observed in men. Additionally, a statistically significant increase in non-supervised manual work at moderate intensity between 3 and 12 months with a non-significant decrease in recreational activities was observed in a subset of women. There were no significant changes in non-supervised activities of the four categories over time in a subset of men. On the other hand, time allocated to non-supervised activities of lower intensity during the first three months decreased significantly in women and returned to similar baseline value at the end of the intervention. Although not statistically significant, participation in higher intensity non-supervised exercises tended to be greater at 3 months and maintained at 12 months for both men and women.

Our study also demonstrated that most of the increase in physical fitness, including CRF, was achieved within the first three months of the intervention. Furthermore, an important finding of the study is that participants maintained their improvements at the end of the intervention even with reduced supervision, which may be explained by an increased participation in higher intensity non-supervised exercises at 3 months and maintained at 12 months. The long-term maintenance of early fitness benefits of exercise are concordant with the results obtained in the RESOLVE Study. Following an intensive residential program including endurance and resistance exercise, the participants in the RESOLVE Study maintained their fitness improvements despite a reduced supervision over a 1-year subsequent follow-up [10]. This is also consistent with our clinical experience in obesity management showing that fitness gains resulting from a first phase of diet-drug intervention are accentuated by a second phase of diet-physical activity supervision [29]. Finally, our results are in agreement with a meta-analytical review of literature emphasizing the relevance of long-term intervention to influence metabolic health [30].

This study is not without limitations. The exercise specialists were instructed to deliver the exercise program in accordance with ACSM and CSEP guidelines targeting a duration of 45–50 min at a given intensity during the first three months. However, they were also instructed to design a personalized program based on the ability and needs of the participants; hence it is not surprising to see the exercise target values were below these guidelines. Personalization is critical in increasing effectiveness of lifestyle interventions in the health system, but has implications for analysis and interpretation. Also, the limited number of subjects who reported on their physical activity habits may have diminished the strength of the statistical analysis. Some non-significant results may be due to this lack of statistical power.

However, other research has suggested that patient motivation leading to improved lifestyle adherence can be enhanced via frequent encounters with health care professionals [8]. This intervention provided very frequent encounters, and therefore provides a baseline for the typical achievable goals among middle-aged patients in primary care practice. The program was a success with a median attendance of 76% of expected exercise and follow-up visits and significant improvements in physical fitness and activity habits. Patient experience data, published elsewhere, also indicated that 63% of patients were totally or very confident they could maintain the physical activity changes [21].

As reported by Jeejeebhoy et al. [18], our intervention induced favorable changes in some components of MetS. This study also showed that there was a reversal of MetS in 19% patients and 42% patients had improvements in at least one component of MetS at 12 months [18]. This agrees with results of numerous detailed controlled studies which have documented beneficial effects of lifestyle programs including exercise and diet on the metabolic profile of individuals with MetS [6, 8, 30–32]. Others have demonstrated concomitant increases in maximal oxygen uptake in response to exercise training [9, 15]. The corollary of the observation that our program induced beneficial effects in a fraction of our participants is that there were also some subjects who responded less favorably to the intervention. In this regard, one of our primary goals was to determine if the high responders in VO_2_ max were also those who displayed the most pronounced improvement in metabolic profiles. The results showed that women exhibiting the most pronounced increase in VO_2_ max were also those who showed the most pronounced decrease in WC. However, this effect was not observed in men. We also found no relationship between changes in VO_2_ max and any other MetS components. Taken together, these observations show that even if the association between variations in aerobic and metabolic fitness remains plausible and interesting, further research is needed to validate this concept. Such research should consider various exercise modalities which might ultimately reveal that the approach used in this study was not sensitive enough to induce MetS changes of sufficient importance to highlight this relationship. In addition, MetS identifies a heterogenous group in health care, some of whom may not benefit from lifestyle interventions.

## Conclusion

In summary, patients with MetS improved physical fitness and physical activity habits over one year through a personalized lifestyle program of diet and exercise in a primary care setting that includes a FP, dietitian and kinesiologist. Changes in CRF were associated with the response of waist circumference to the intervention in women.

## Data Availability

The datasets used and analysed during the current study are not available for sharing.

## Appendix

As described by Jeejeebhoy et al.^17^, we excluded patients who were not able to participate in the supervision for medical, safety or logistic reasons. Specifically, we considered the following inclusion and exclusion criteria:

**Inclusion Criteria:** Adult Patients identified by their family doctor as having the metabolic syndrome.

- Age > 18 y

Metabolic syndrome is defined as having 3 out of 5 of the following criteria:

- Fasting Blood Glucose > 5.6 mmol/L or receiving pharmacotherapy
- Blood Pressure of > 130/85 mmHg or receiving pharmacotherapy.
- Triglyceride of > 1.7 mmol/L or receiving pharmacotherapy
- HDL-C < 1.0 mmol/L Males and < 1.3 mmol/L females
- Abdominal circumference as determined by a pre-specified technique:
- Europids, Whites, sub-Saharan Africans, Mediterranean, middle east (Arab) > 94 cm
  Males, 80 cm Female.
- Asian and South Central Americans > 90 cm males and 80 cm females
- US and Canadian Whites > 102 cm males, 88 cm females.

**Exclusion Criteria:** Patients who, for medical reasons, were unable to exercise or have a life-limiting illness that such a longitudinal study would not be appropriate were excluded. Subjects meeting any one of the following exclusion criteria were not enrolled into the study.

1. Inability to speak, read or understand English and/or French for the Laval University participants.

2. Having a medical or physical condition that makes moderate intensity physical activity (like a brisk walk) difficult or unsafe.

3. Diagnosis of Type 1 diabetes mellitus.

4. Type 2 diabetes mellitus if any one of the following are present.

a. Proliferative diabetic retinopathy.

b. Nephropathy (serum creatinine > 160 μmol/L).

c. Clinically manifest neuropathy defined as absent ankle jerks.

d. Severe hyperglycemia (FBS > 11 mmol/L).

e. Peripheral vascular disease.

5. Significant medical co-morbidities, including uncontrolled metabolic disorders (e.g., thyroid, renal, liver), heart disease, stroke and ongoing substance abuse.

6. Clinically significant renal failure.

7. Diagnosis of psychiatric disorders (cognitive impairment) that would limit adequate informed consent or ability to comply with study protocol.

8. Diagnosis of cancer (other than non-melanoma skin cancer) that was active or treated with radiation or chemotherapy within the past 2 years.

9. Diagnosis of a terminal illness and/or in hospice care.

10. Pregnant, lactating or planning to become pregnant during the study period.

11. Investigator discretion for clinical safety or protocol adherence reasons.

12. Chronic inflammatory diseases.

13. Body Mass Index > 35 kg/m^2^.

## Abbreviations

ACSM: American College of Sports Medicine
BMI: Body mass index
CHANGE: Canadian Health Advanced by Nutrition and Graded Exercise
CRF: Cardiorespiratory fitness
CSEP: Canadian Society for Exercise Physiology
CV: Cardiovascular
CVD: Cardiovascular disease
FD: Family doctor
HDL: High-density lipoprotein
MetS: Metabolic syndrome
MVPA: Moderate to vigorous physical activity
NHANES: National Health and Nutrition Examination Survey
RD: Registered dietitian
SE: Standard error
T2DM: Type 2 diabetes
VO_2_max: Maximal oxygen consumption
WC: Waist circumference
